# Supplementary figures and images for: Development of a novel embryonic germline gene-related prognostic model of lung adenocarcinoma
Source: PeerJ. 2021 Oct 21;9:e12257. doi: 10.7717/peerj.12257 (PMC8542372; doi:10.7717/peerj.12257)

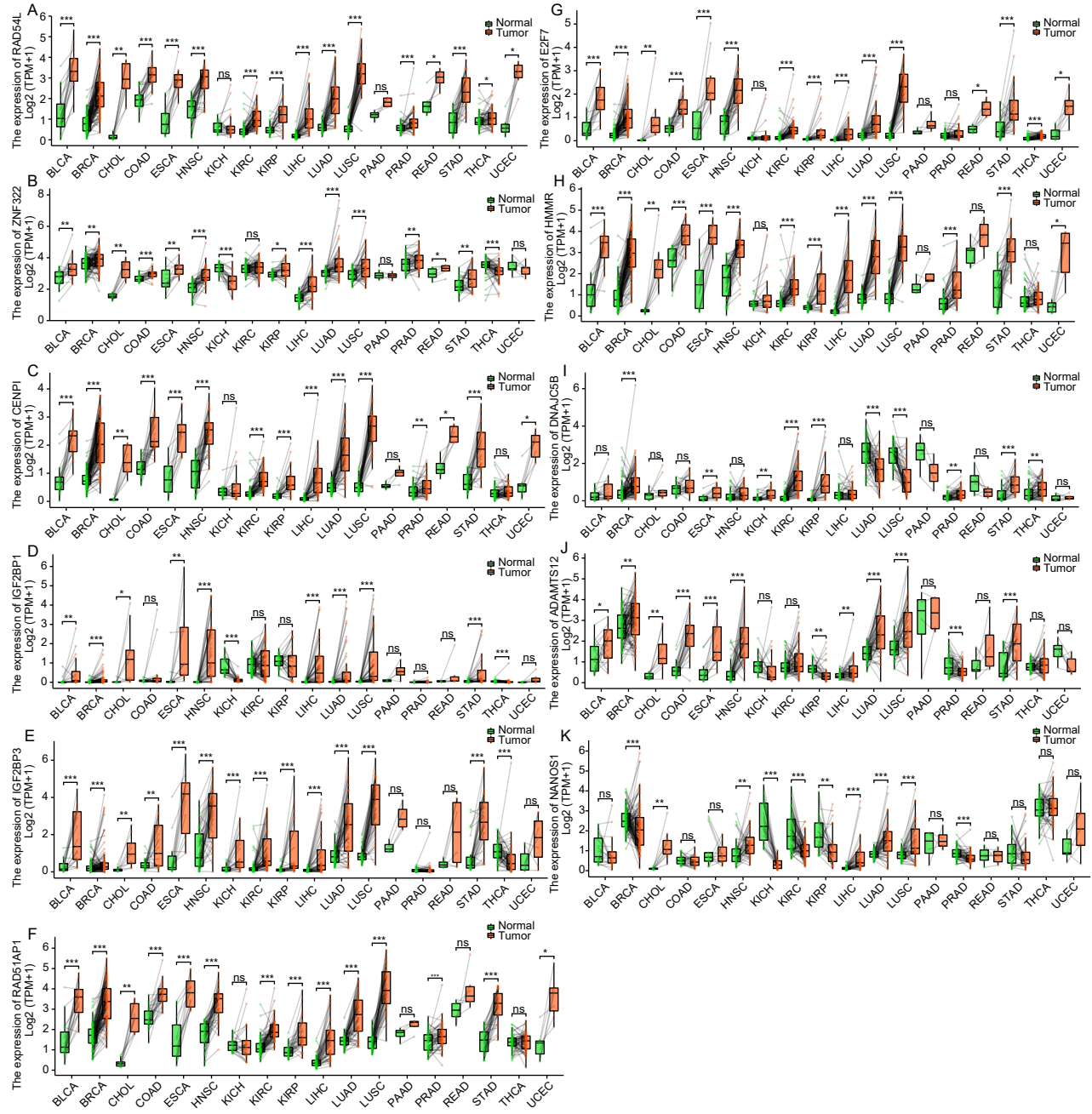

Supplement: Supplemental Information 4 — The differential expression analyses of RAD54L(A), ZNF322(B), CENPI(C), IGF2BP1(D), IGF2BP3(E), RAD51AP1(F), E2F7(G), HMMR(H), DNAJC5B(I), ADAMTS12(J) and NANOS1(K) between tumor and paired adjacent normal tissues. *, p < 0.05; **, p < 0.01; ***, p < 0.001; ns, not significant. DEGGs: differentially expressed embryonic germline genes; BLCA: Bladder Urothelial Carcinoma; BRCA, Breast invasive carcinoma; CHOL, Cholangiocarcinoma; COAD, Colon adenocarcinoma; ESCA, Esophageal carcinoma; HNSC, Head and Neck squamous cell carcinoma; KICH, Kidney Chromophobe; KIRC: Kidney renal clear cell carcinoma; KIRP, Kidney renal papillary cell carcinoma; LIHC, Liver hepatocellular carcinoma; LUAD, Lung adenocarcinoma; LUSC, Lung squamous cell carcinoma; PAAD, Pancreatic adenocarcinoma; PRAD: Prostate adenocarcinoma; READ, Rectum adenocarcinoma; STAD: Stomach adenocarcinoma; THCA, Thyroid carcinoma; UCEC: Uterine Corpus Endometrial Carcinoma. [file peerj-09-12257-s004.pdf]
